# *In* *silico* modelling of the effect of pyloric intervention procedures on gastric flow and emptying in a stomach with gastroparesis

**DOI:** 10.1098/rsif.2023.0567

**Published:** 2024-01-24

**Authors:** Sharun Kuhar, Jung-Hee Seo, Pankaj J Pasricha, Rajat Mittal

**Affiliations:** ^1^ Department of Mechanical Engineering, Johns Hopkins University, Baltimore, MD 21218, USA; ^2^ Mayo Clinic, Phoenix, AZ 85054, USA; ^3^ Department of Medicine, Johns Hopkins University, Baltimore, MD 21205, USA

**Keywords:** computational fluid dynamics, gastroparesis, stomach model, pyloroplasty, bile reflux, surgical intervention

## Abstract

Pyloric interventions are surgical procedures employed to increase the gastric emptying rate in gastroparesis patients. In this study, we use an *in silico* model to investigate the consequences of pyloric intervention on gastric flow and emptying for two phenotypes of gastroparesis: antral hypomotility and decreased gastric tone. The transpyloric pressure gradient predicted by the *in silico* model, based on viscous fluid flow equations, is compared against *in vivo* measurements. Both phenotypes exhibit a similar pre-procedural emptying rate reduction, but after pyloric surgery, antral hypomotility case with preserved gastric tone shows significant improvements in emptying rates, up to 131%, accompanied by bile reflux from the duodenum into the stomach. Conversely, severely reduced gastric tone cases exhibited a post-procedural reduction in the net emptying rate due to the relatively larger bile reflux. In cases with a combination of antral hypomotility and reduced gastric tone, post-procedural improvements were observed only when both conditions were mild. Our findings highlight the pivotal role of the relative increase in pyloric orifice diameter in determining post-operative emptying rates. The study suggests a possible explanation for the selective response of patients toward these procedures and underscores the potential of *in silico* modelling to generate valuable insights to inform gastric surgery.

## 1. Introduction

**Figure 1.**
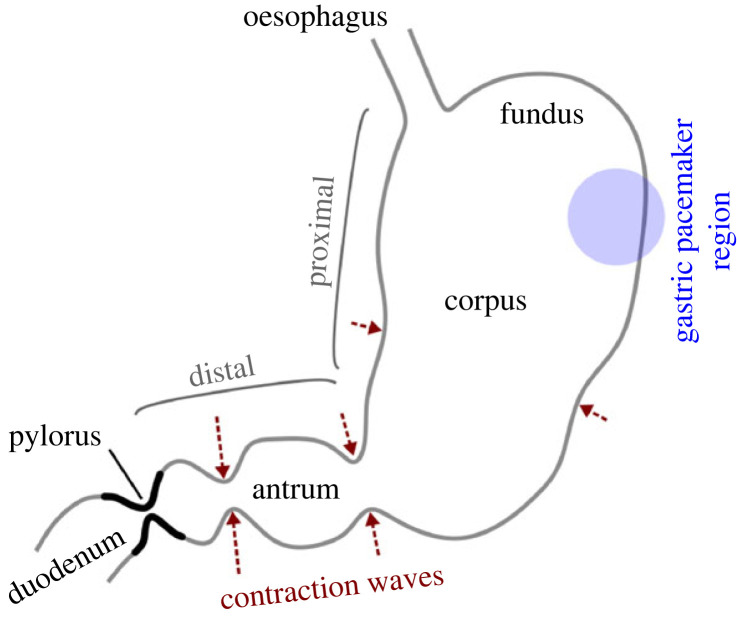
A schematic of the stomach.

The stomach acts as a storage, mixer, grinder, sieve and chemical processor of the ingested meals. It has three main regions—fundus, corpus and antrum (figure 1). The fundus accommodates the incoming meal while maintaining constant pressure inside the lumen. The corpus is the main body of the stomach that houses the pacemaker where small amplitude peristaltic contractions originate and move towards the pylorus, growing in amplitude as they reach the antrum [1]. The pylorus connects the stomach to the duodenum, which is the initial segment of the small intestine. The pyloric orifice opens for a brief period as each contraction moves across the antrum, allowing liquid and small-sized particles to empty; the orifice then constricts as the contraction reaches the terminal antrum forcing the larger particles to be propelled back into the stomach for continued trituration. Both the gastric muscle tone of the fundus and the antral contraction waves (ACWs) play a role in emptying the stomach’s contents, with the former being the primary mechanism for emptying and the latter for the grinding and subsequent emptying of solid food particles [2,3]. Each of these physiological responses to a meal may contribute to the global rate at which the stomach empties into the intestines, a measure that is widely used clinically and is directly or indirectly linked to many disorders like—dumping syndrome [4], gastroparesis (idiopathic or diabetic) [5] and Parkinson’s disease [6].

Gastroparesis, the focus of this study, is defined as the reduced rate of gastric emptying in the absence of any mechanical obstruction. While the condition is often idiopathic [7], it is often associated with diabetes mellitus or post-surgical complications of gastric surgeries [8]. It is characterized by symptoms including nausea, vomiting, early satiety, bloating and upper abdominal pain. Gastroparesis can be diagnosed by ruling out any mechanical obstruction and quantifying the delay in gastric emptying using scintigraphy or other emerging techniques [5,9]. The disorder impacts the quality of life, and sometimes patients may need to be hospitalized due to severe pain, retching or discomfort. The number of hospital admissions associated with gastroparesis has increased over four times since 1997 along with a significant rise in inpatient costs [10]. The current treatment options are also not meeting the needs of the patients. In 2017, a survey found that 27% of the patients were ‘somewhat dissatisfied’ and 33% of them were ‘dissatisfied’ with the available treatments [11].

Since there is no cure, the treatment for gastroparesis focuses on relieving symptoms [12]. A recent guideline published by field experts describes the sequelae for clinical management of the disorder [5]. Dietary modifications and pharmacological options are the first recommendations, but a large fraction of the patients [13] do not respond to these treatments. Such patients are labelled refractory and are prescribed surgeries such as the pyloric intervention procedures. These surgeries treat the pylorus as a therapeutic target based on the theory that ‘pylorospasms’, or unusual resistance to emptying by the pylorus, may be an important mechanism in the pathogenesis of delayed gastric emptying in at least a subset of patients [14,15]. The two primary pyloric intervention procedures are surgical pyloroplasty and pyloromyotomy (gastric per-oral endoscopic myotomy or GPOEM), with the latter being a novel and more attractive option as it is less invasive and has comparable outcomes to pyloroplasty [16]. These procedures enlarge the pylorus and disrupt its sphincter function, and are based on the notion that a patent pylorus should empty the stomach faster [12,17,18]. Other pyloric intervention procedures, like botulinum toxin injection or pyloric stent, also use the same rationale but have not been proven to be effective in controlled trials [5].

Multiple studies report a clinical success rate of pyloroplasty or pyloromyotomy in the range of 60–80% [7,12,16,19,20]. Although impressive, this still represents a relatively large rate of failure and raises the important question of whether there are better ways to select patients for this procedure [13]. Recent studies have discussed potential strategies for predicting the response to these surgeries [14], but an intrinsic limitation to success is the lack of a clear understanding of the effects of pyloric intervention in different pathophysiological subgroups, e.g. those with antral hypomotility or altered fundic accommodation. Furthermore, these surgeries may sometimes lead to bile reflux from the duodenum into the stomach [21–23] which may not only damage gastric mucosa but result in reflex-driven alterations in gastric motility. Newer paradigms for approaching these important questions are therefore needed.

Computational modelling of gastric digestion is gaining interest in recent times and is a useful tool to complement experimental observations. Since the first two-dimensional modelling studies in 2004 [24], multiple three-dimensional models have been developed to investigate the emptying rate, mixing, recirculation and effect of posture [25–29]. In recent years, computational models of the stomach are starting to incorporate new complexities to address more specific questions. Ishida *et al.* studied the effect of changes in the timing of pylorus opening relative to the incoming contractions, which might be disrupted due to surgery [30]. Others have incorporated acid secretion and solid food into their models to study pH distribution and bolus stacking [31,32]. Some models have also incorporated the functionality of the stomach muscle fibres and their orientation into their model [33]. Computational models can help fill the gaps and shed light on mechanisms that would otherwise be hard to explore with experimental techniques. There have been attempts to model the effect of hypomotility, which is one of the mechanisms observed in gastroparesis, on stomach digestion [34]. The use of these models to examine the effect of gastric surgeries, the subject of the current work, has, however, not yet been attempted.

In this study, we use a high-fidelity computational model of the human stomach called ‘StomachSim’ [35,36] to investigate these different gastroparesis phenotypes and the effect of pyloric intervention procedures on restoring gastric emptying. We mimic a healthy case, and two gastroparesis phenotypes—one due to hypomotility and the other due to decreased gastric tone. We then investigate the differences in the flow and emptying of the contents before and after pyloric intervention, referred to as pre- and post-operative cases, respectively. In the past, we have used StomachSim to examine the effects of gastroparesis on the dissolution of an oral pill [35,36] and on the hydrolysis of a liquid meal [34]. In this study, we model the changes in gastric flow phenomenon for gastroparesis cases before and after pyloric intervention procedures. The computational model allows us to study the competing effects of the two mechanisms and understand possible reasons behind the cases not responding to these surgeries.

## 2. Methodology

### 2.1. The stomach model

**Figure 2.**
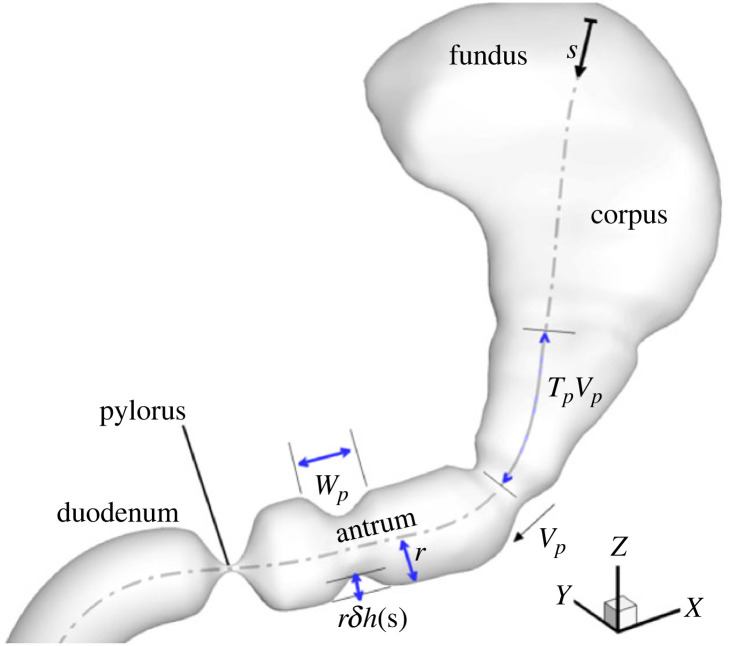
The stomach model is shown with the different regions and the contraction parameters. ‘*s*’ is the distance along the centerline of the stomach, *T*_*p*_ is the time elapsed between consecutive contraction waves, and *V_p_* and *W_p_* are the velocity and width of the contractions, respectively. ‘*r*’ is the distance of any point on the walls before the contraction and ‘*δ*’ is a constant fraction corresponding to the amplitude of the contraction waves. The function *h*(s), with a value of 0 in the mid-corpus and 1 in the antrum, modifies the amplitude from one region to another.

The stomach geometry and motility model used in this study are described here in brief, since a detailed description can be found in our previous work [34]. The stomach geometry was segmented from magnetic resonance imaging (MRI) data of a 34-year-old male available in the Virtual Population Library [37]. As is evident from the tubular shape of the antrum, this geometry corresponds to a small volume of ingested food, although we do not expect the simulation outcomes to change significantly if an enlarged stomach with larger volume of ingested meal is used instead. The stomach wall contractions are modelled as sinusoidal deformations of the walls toward the centreline of the lumen, increasing in amplitude as they move toward the antrum (figure 2). Right before the contractions arrive at the pylorus, they terminate in a high-amplitude collapse of the walls—an event termed terminal antral contraction (TAC). The pylorus constricts before the end of this terminal collapse, causing the trapped contents to be ejected back with force. This mechanism enhances mixing and accelerates the breakdown of solid food particles. All variations in the amplitude of the contractions, from corpus to antrum as well as due to the TAC, are incorporated into the model through a function whose value changes along the centreline.

Several experimental studies have measured the kinematics of gastric contractions but antral mobility is found to be quite sensitive to food properties [38,39]. We focus here on the case of a low-calorie liquid meal where, based on several imaging studies [40–44], a frequency of 2.6−3 waves min^−1^ (i.e. *T*_*p*_ = 20−23 s) and a mean amplitude in the range of 34–40% of the lumen is found to be typical. The mean speed of contraction waves, however, shows a larger variation, ranging from 1.6 to 2.8 mm s^−1^. In this study, we used a frequency of 3 waves min^−1^ (*T*_*p*_ = 20 s), an amplitude of 40% (*δ* = 0.4) and a wave speed of 2.3 mm s^−1^ (=*V*_*p*_).

### 2.2. Flow model

The above-described stomach geometry and kinematics are immersed in a three-dimensional Cartesian grid of 25M grid points and the incompressible Navier–Stokes equations are solved for the gastric contents,

2.1 ∇⋅u=0

and

2.2 $$\rho \left(\frac{\partial u}{\partial t}+u\cdot \nabla u\right)=-\nabla p+\mu \nabla^{2}u+\rho g,$$

where u is the flow velocity, *p* is the pressure, and *ρ* and *μ* are the fluid density and viscosity, respectively. For the normal stomach, the gastric contents were assumed to have the properties of water (*ρ* = 1000 kg m^−3^ and *μ* = 1 mPa s), but we have also examined the effect of viscosity on the flow features and fundus pressure. The time-step and grid convergence studies and the details of the numerical methods employed can be found in our previous work [34]. We also describe in that work that the flow field becomes periodic after three contraction cycles. Since the contractions originate every 20 s and move towards the antrum, the shape of the stomach at a given time is the same as it was 20 s ago. Consequently, after a period of initial transience, the flow inside the lumen also repeats with the same time period, i.e. u(x,t)=u(x,t+τ) where *τ* = 20 s. Using the same approach, all results presented in this work correspond to the duration after the flow has become periodic.

#### 2.2.1. Boundary conditions and gastric tone model

**Figure 3.**
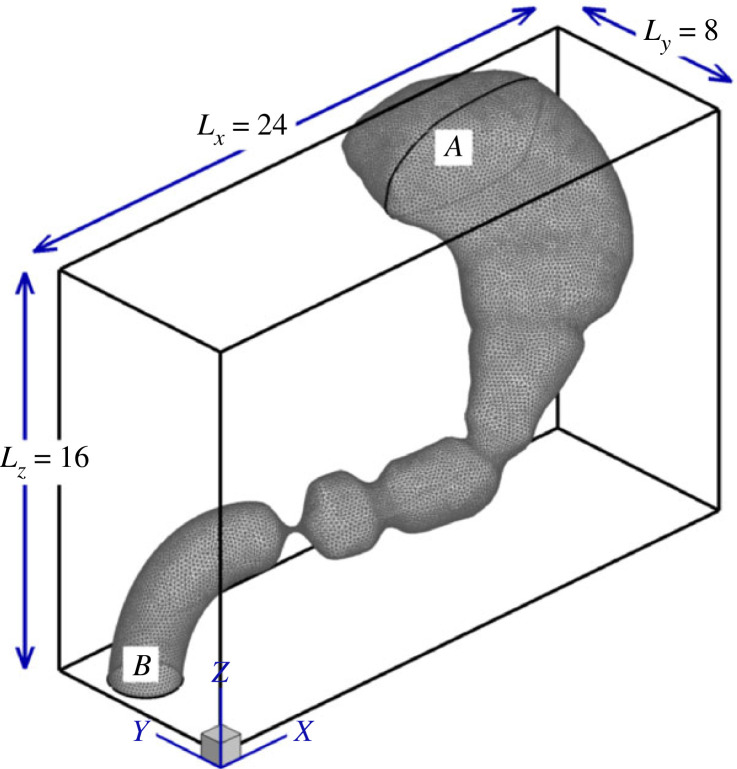
The triangulated surface mesh is immersed in an outer Cartesian mesh with an open fundus at the top (labelled A) and an open duodenum at the bottom (labelled B).

A no-slip boundary condition ($u=u_{\mathrm{wall}}$) along with a zero gradient pressure boundary condition ($\nabla p\cdot \hat{n}=0$) is applied on the stomach walls. The stomach model has two openings where additional boundary conditions are needed—at the fundus and at the duodenum (labelled ‘A’ and ‘B’ in figure 3, respectively). All recent computational models of the stomach incorporate antral wall motility [45]; however, gastric tone, which is the primary driver for emptying, has not been incorporated in most models. In the current study, we incorporate the effect of gastric tone and thereby include the two key biomechanical mechanisms for the gastric phase of digestion—antral motility and gastric tone. As shown later, this not only enhances the anatomical fidelity of the model, it also allows us to examine two different phenotypes of gastroparesis. Furthermore, the same feature of the model can also be used to study the effects of fundus relaxing drugs or the consequences of proximal gastric vagotomy, both of which reduce gastric tone.

In a postprandial stomach, the fundus applies a nearly constant pressure (i.e. the gastric tone) on the stomach contents that provides the impetus for gastric emptying, but it also allows for volume accommodation of the stomach contents, including the volume changes associated with antral contractions. We mimic this phenomenology as a fundic ‘piston’ with finite inertia at the fundus opening (labelled ‘A’) that is driven inwards by a constant external pressure *p*_*o*_. The motion of such a piston is governed by the following equation:

2.3 $$m_{\;p}A\frac{dv_{\;p}}{dt}=p_{o}A-\int_{A}p\;dA\;⟹\;\frac{dv_{\;p}}{dt}=\frac{\;p_{o}-{\bar{\;p}}_{A}}{m_{\;p}},$$

where *m*_*p*_ is the mass per unit area of the piston, *v*_*p*_ is the piston velocity, *A* is the area of the piston, and ${\bar{\;p}}_{A}$ is the average pressure on the fluid side of the piston. Since the fluid adjacent to the piston moves at the velocity of the piston, the above equation provides the following prescription for the rate-of-change in time of the normal flow velocity (*u*_*n*_) at the piston,

2.4 $$\frac{du_{n}}{dt}=\frac{\;p_{o}-{\bar{\;p}}_{A}}{m_{\;p}},$$

which can be discretized using an explicit scheme to provide a value of velocity at the boundary. For pressure, we specify a zero gradient at the fundus opening, i.e. ∂*p*/∂*n* = 0.

We note that in the model of fundic tone above, *p*_*o*_, which represents the pressure exerted by the fundus, affects the flux at the fundic inlet, and consequently at the duodenal outlet. The fundic pressure *p*_*o*_ can therefore be prescribed to not just achieve the desired emptying rate into the duodenum, but also to model the effect of decreased gastric tone. The parameter *m*_*p*_ represents the inertia in the boundary condition and this parameter controls the numerical stability of the system—too low a value results in a stiff system that leads to numerical instability. In the current study, *m*_*p*_ = 1000 was chosen for all cases after testing the stability of the model for the case with the strongest motility and lowest viscosity. With this chosen value, the results of the model were relatively insensitive to the choice of this parameter—a 400% change in the value of *m*_*p*_ led to a less than 5% change in the emptying rate for a fixed value of *p*_*o*_.

At the duodenal end (labelled ‘B’), a Neumann velocity boundary condition ∂u/∂n=0 is applied, which conserves the mass flux through the stomach. The absolute value of pressure at the duodenum is specified as *p* = 0. This implies that all pressure values in the simulations are to be considered as pressure differences with respect to the duodenum.

### 2.3. Modelling gastroparesis

The reduction in emptying rate in gastroparesis can arise due to several different mechanisms [12]. At the organ level, it can manifest via (i) decreased gastric tone—the proximal stomach does not apply enough pressure on the contents; (ii) antral hypomotility—the peristaltic contractions of the walls are not strong enough; (iii) reduced pyloric relaxation—the pylorus offers excessive resistance to the gastric contents. Although the underlying basis of these changes is poorly understood, gastric dysrhythmia and impaired electromechanical coupling may perhaps drive many of them, particularly wall contractions.

**Figure 4.**
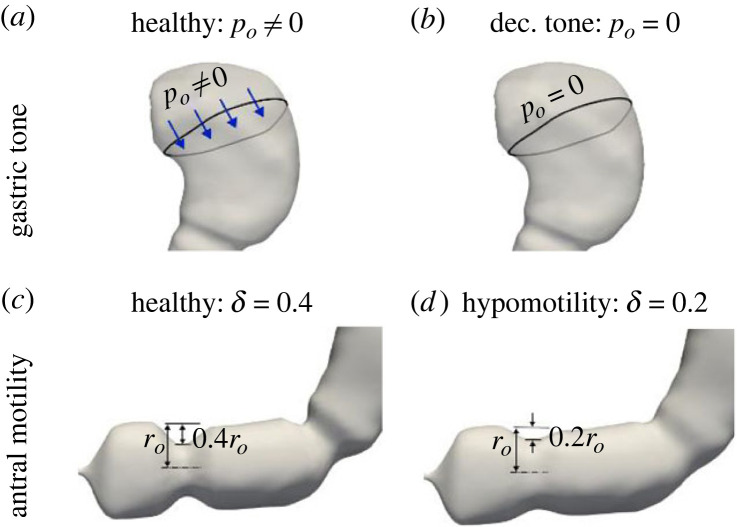
Two phenotypes of gastroparesis are modelled: decreased gastric tone, incorporated by changing the fundic pressure (*a*) versus (*b*), and antral hypomotility, modelled as a reduction in the amplitude of contractions (*c*) versus (*d*)

For the healthy stomach, the fundic pressure *p*_*o*_ is set equal to 0.04 and 0.5 mm Hg for the low-viscosity meal (1 mPa s) and high-viscosity meal (50 mPa s), respectively, so as to achieve an experimentally observed emptying rate of 4.3 ml min^−1^ for low-calorie liquid meals [46,47]. For modelling gastroparesis due to poor gastric tone, *p*_*o*_ is set to a value of zero. For antral hypomotility cases, the amplitude of stomach wall contractions (*δ*; see figure 2) was lowered to half their normal value as observed in patients [48]. The changes corresponding to these cases are also described in figure 4. The reduced pyloric relaxation case was not considered for this study, since its effect on gastric emptying is trivial in the context of the current model.

### 2.4. Pyloroplasty

**Figure 5.**
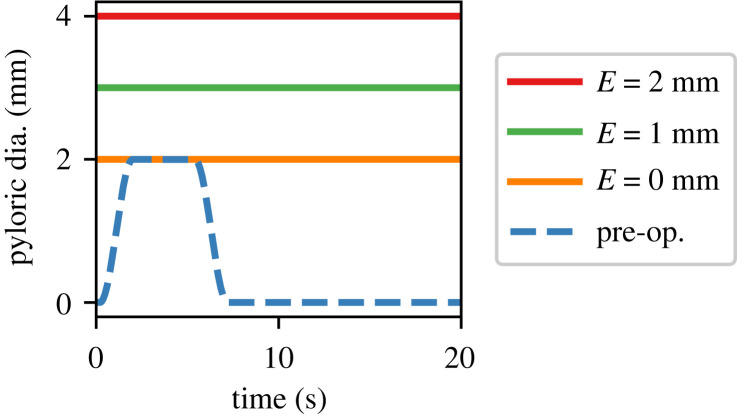
Pyloric intervention procedures are modelled as an enlarged pyloric orifice whose sphincter function is disrupted and stays open throughout the cycle. Three cases of post-operative enlargement, *E*, are considered E=0,1, and 2 mm.

As described earlier, in pyloroplasty or in pyloromyotomy, the pyloric orifice is enlarged and the closure of the pyloric sphincter is disrupted [12,17,18]. While in pre-operative cases the pylorus is allowed to close completely and to open to a maximum diameter of 2 mm, in the post-operative cases we limit the closure of the pyloric orifice to a value larger than zero, thereby keeping the pylorus permanently patent. The orifice measurements after the surgery are not readily available in the literature because it is challenging to measure its size in the post-prandial state. However, studies suggest that these procedures result in an enlargement of 1.2–2 mm in the orifice diameter [49,50]. In this study, we consider three post-operative diameter enlargements, *E*, of 0, 1 and 2 mm to study the effect of enlargement on the efficacy of the surgery (figure 5). In keeping with *in vivo* studies [51], the antral wall contractions in the post-operative cases are kept the same as the pre-operative conditions.

## 3. Results

The *in silico* model is used to simulate a healthy case (control) and several gastroparesis cases before and after the surgery (referred to as pre-operative and post-operative cases, respectively). In the first section (§3.1), we present a comparison of our model predictions against available *in vivo* data while also showing the effect of food viscosity on the flow field. For the remaining sections, the viscosity is kept fixed (*μ* = 1 mPa s). In the two subsequent sections (§§3.2 and 3.3), the flow and the emptying rate are compared for the healthy case and gastroparesis cases arising from hypomotility and from decreased gastric tone. After that (§3.4), we study the effect of the varying degrees of severity of these mechanisms and the differences in the results if each mechanism occurs separately or concomitantly. In the last section (§3.5), the consequences of different pyloric enlargements are compared.

### 3.1. Transpyloric pressure gradient: comparison with *in vivo* data

Measuring flow velocities *in vivo* is challenging and pressure remains the primarily documented variable in *in vivo* studies. However, finding data to compare with computational models continues to be a challenge today because of the limited availability of the required measurements and the high variability in those measurements with respect to the different meals used in the studies and subject-to-subject differences.

**Figure 6.**
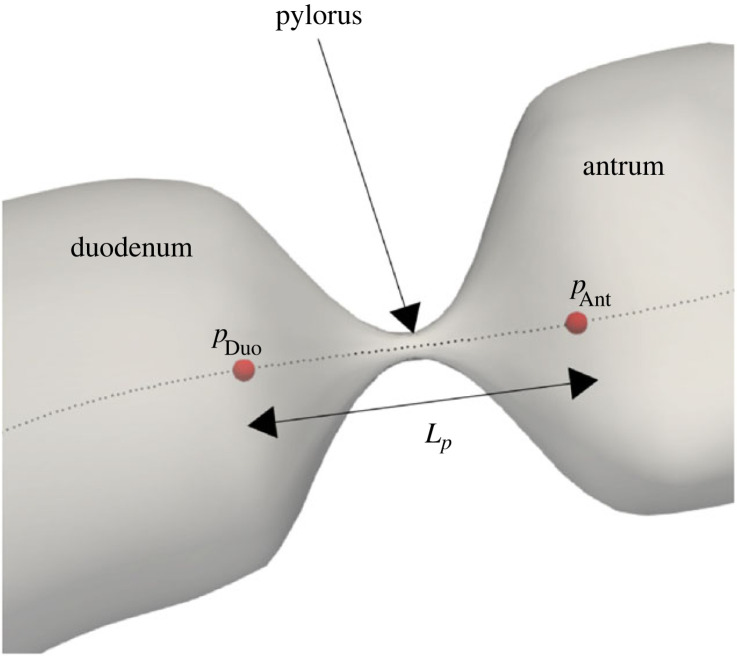
Location of antral and duodenal points used to measure transpyloric pressure gradient as Δ*p*/*L* = (*p*_Ant_ − *p*_Duo_)/*L*_*p*_.

Pressure measurements by endoscopic manometry are the gold standard among researchers. However, comparing pressure values with computational models requires a common reference between the studies involved and the authors could find one study that reported pressure gradients instead of absolute pressure measurements. Indireshkumar *et al.* [52] reported that the transpyloric pressure gradient averaged over seven subjects was 0.3 ± 0.2 mm Hg cm^−1^ for a meal of unknown viscosity entering the duodenum at a rate of 2.1 kcal min^−1^. This gradient was measured as (*p*_Ant_ − *p*_Duo_)/*L*_*p*_, where *p*_Ant_ is the pressure measured by the manometer port in the antrum and *p*_Duo_ is that in that duodenum, and *L*_*p*_ is the distance between the two ports. Following the same procedure (figure 6), we extracted the pressure gradient for different viscosities in our model averaged over the duration for which the pylorus is open and found that the values for high-viscosity meals predicted by the current model are in line with the range provided by the *in vivo* study (table 1).

**Table 1.** Comparison of transpyloric pressure gradient for different food viscosities with *in vivo* data.

study	Δ*p*/*L* (mm Hg cm^−1^)
present (*μ* = 1 mPa s)	0.02
present (*μ* = 50 mPa s)	0.25
Indireshkumar *et al.* [52]	0.3 ± 0.2

**Figure 7.**
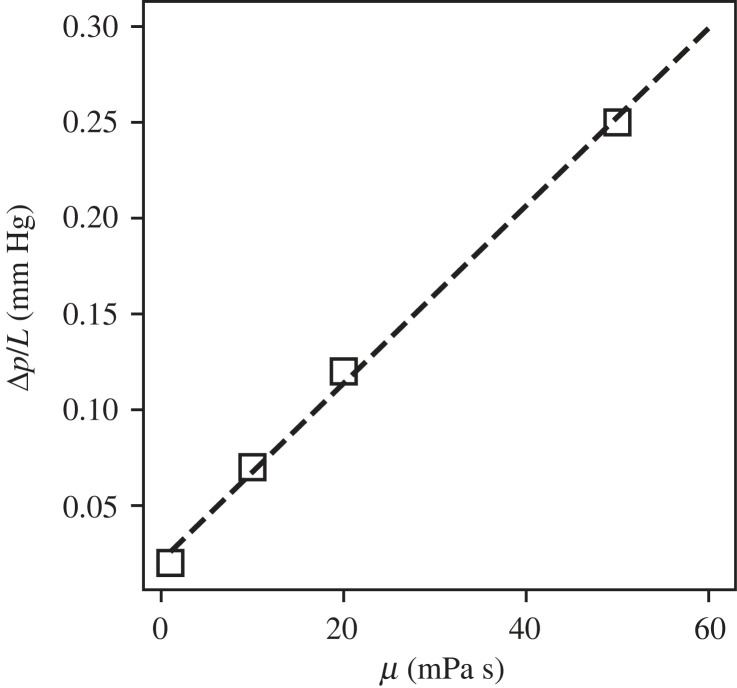
For the same emptying rate, the transpyloric pressure gradient increases linearly with food viscosity.

**Figure 8.**
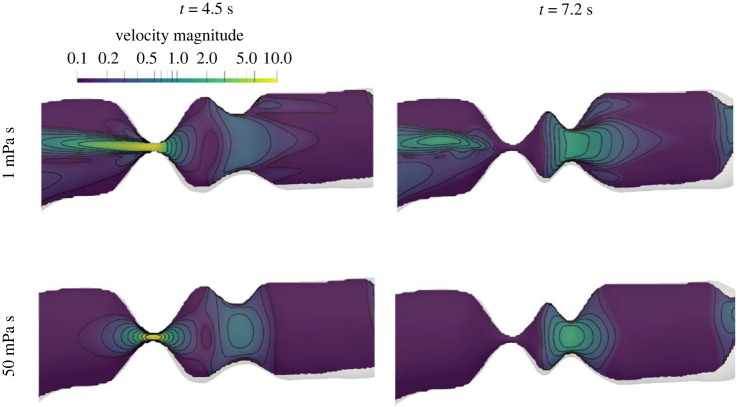
Velocity magnitudes (cm s^−1^) at a cross-section through the antrum at two instances—when pylorus is fully open (4.5 s) and right after its closure (7.2 s)—for different food viscosities.

Furthermore, we found that the transpyloric pressure gradient increases linearly with food viscosity for the same emptying rate, as shown in figure 7. The assumption of the emptying rate staying the same is not unfounded—Marciani *et al.* [47] showed that a rise in viscosity by a factor of 500 only decreased the emptying rate by a factor of 1.2 for the same caloric density meal. Hence, the linear dependence highlights that the stomach adjusts the gastric tone according to the viscosity of the contents. The flow field also becomes much weaker and localized with high-viscosity meals as highlighted in figure 8. The retrograde and pyloric jet lengths are shorter for higher food viscosity, and, consequently, the mixing of contents is slower for viscous meals.

Pyloric flow resistance is another related parameter that is found in a few *in vivo* studies. It is defined as the mean pressure difference across the pylorus divided by the mean flow rate. In this study, the pyloric flow resistance was 0.1 mm Hg ml^−1^ s for 1 mPa s meal viscosity, and 2.4 mm Hg ml^−1^ s for 50 mPa s meal viscosity. One study on pigs reported a pyloric resistance of 4.6 mm Hg ml^−1^ s using a saline meal. Experiments done on dogs employing solid meals blended to a liquid of viscosity 1630 mPa s report a pyloric flow resistance of 6.8 and 8.3 mm Hg ml^−1^ s in different studies [53,54]. It is difficult to compare the present study with these measurements because these experiments were done on animals and employed different meals, but the current numbers seem to be of a reasonable order of magnitude.

### 3.2. Flow inside the stomach

**Figure 9.**
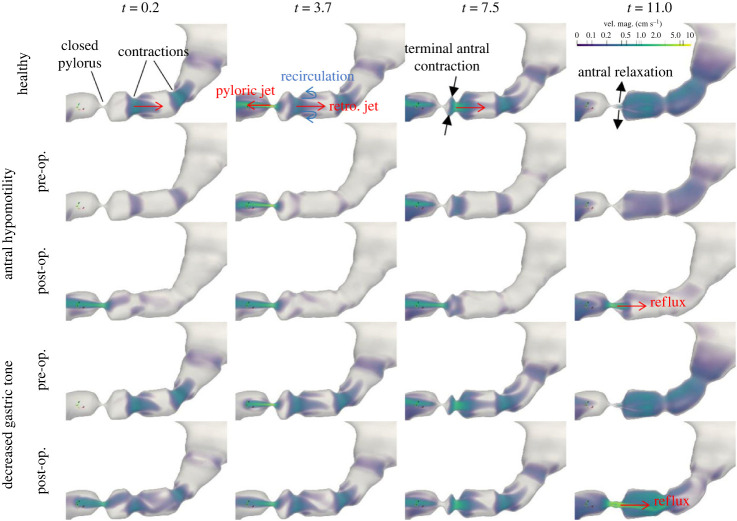
A comparison of velocity magnitudes inside the stomach for one contraction cycle for *μ* = 1 mPa s. Each contraction propagates towards the pylorus generating a retrograde jet in its wake. As the contraction comes closer, the pylorus opens up and the contents empty via the pyloric jet. Then the pylorus closes as the contraction terminates with a large amplitude (terminal antral contraction) propelling the contents back into the stomach via a strong retrograde jet. The collapsed walls relax and the whole process repeats as the next contraction approaches. The post-operative cases (*E* = 2 mm) demonstrate a permanent pyloric jet that reverses the direction during the relaxation phase and leads to reflux.

Figure 9 shows the flow inside the stomach for healthy, antral hypomotility and decreased gastric tone cases. The time sequence shows the propagation of a contraction wave toward the pylorus. The pylorus opens up and allows contents to empty but closes before the contraction reaches the orifice. This is followed by a strong collapse of the walls, also known as terminal antral contraction (TAC), as the contraction reaches the end of the stomach. After TAC, the antrum relaxes and the whole process repeats every 20 s as the subsequent contraction approaches the pylorus in a similar fashion. It is evident that the antrum has a lot more flow activity as compared with the fundus and the corpus, which are relatively quiescent. Regions highlighted by velocity magnitude show three key flow features—a retrograde jet in the wake of the contractions, an annulus of recirculation region near the walls trailing the contraction, and the pyloric jet.

The flow inside the stomach is driven by the wall contractions as well as by the pressure applied by the proximal stomach on the contents. Owing to the lower amplitude of wall contractions in the hypomotility case, the retrograde jet and the recirculation region are significantly weaker as compared with the healthy case (see figure 9, rows 1 and 2), alongside some reduction in the pyloric jet emptying. The decreased gastric tone case retains antral motility, thereby exhibiting flow activity similar to the healthy case in the antrum (rows 1 and 4), but the reduced pressure leads to a much weaker pyloric jet, even weaker than the antral hypomotility case. After pyloric enlargement, i.e. post-operative cases, a more prominent pyloric jet empties contents throughout the cycle for both phenotypes (rows 3 and 5 in column 2). Post-operative cases also show reflux from the duodenum into the stomach during the relaxation phase after the TAC (rows 3 and 5 in column 4) because of the increasing volume of the antrum and the permanent patency of the pylorus. The decreased tone case shows a more significant amount of reflux as compared with the hypomotility case, because the latter undergoes a smaller volume change in the antrum due to weaker contractions. These comparisons are quantified in the next section where we focus on the contents emptied through the pylorus.

### 3.3. Gastric emptying

**Figure 10.**
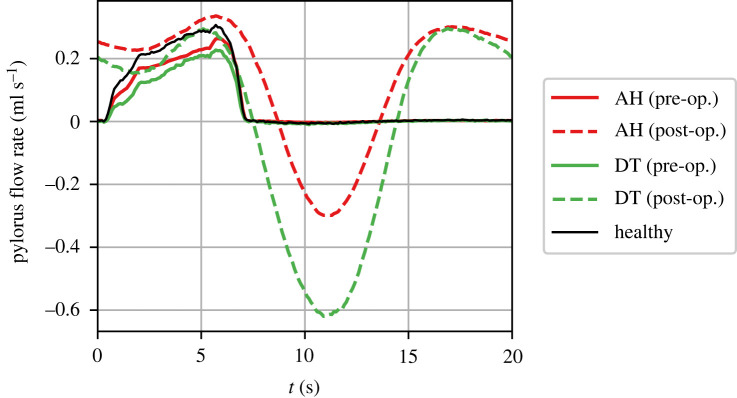
Flow rate through the pylorus from the stomach into the duodenum over one contraction cycle. Antral hypomotility (AH), as well as decreased tone (DT), both lead to a slower emptying rate relative to the healthy case. However, after pyloric intervention with an enlargement of *E* = 2 mm, the two conditions show different amounts of reflux rates and improvement in the emptying rates.

Figure 10 shows the flux of contents across the pylorus over one cycle of contractions. Before the enlargement of the pylorus, the trans-pyloric flux for both gastroparesis cases falls short of the healthy case, indicating reduced emptying. After the procedure (with an enlargement of *E* = 2 mm), the forward flux is increased over the duration of the cycle but there now appears significant reverse flow, i.e. reflux. The reflux is caused by the relaxation of the antrum with the pylorus maintained in a permanently open position as a result of the pyloric intervention. The intra-gastric pressure resists this reverse flow and the amount of reflux is determined by the balance between the opening size of the pylorus, the magnitude of antral relaxation and the pressure applied by the proximal stomach. Consequently, the preserved gastric tone in the hypomotility case leads to less reflux as compared with the decreased tone case. In other words, the decreased tone case cannot apply the necessary pressure to resist the reverse flow and leads to large amounts of reflux.

### 3.4. Reduced gastric tone versus antral hypomotility

**Figure 11.**
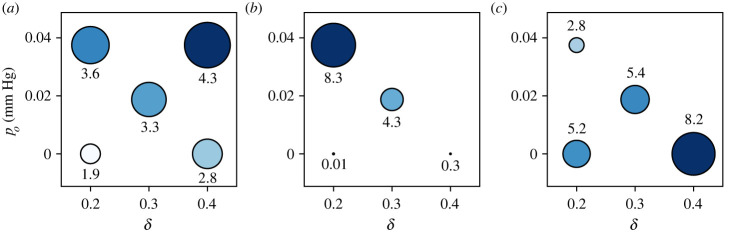
A comparison of different gastroparesis cases on the motility-tone (*δ* − *p*_*o*_) plane before and after the surgery with an enlargement of *E* = 2 mm with food viscosity *μ* = 1 mPa s. (*δ* = 0.4, *p*_*o*_ = 0.04 mm Hg^−1^) corresponds to the healthy case, and all the other cases are different combinations of degrees of antral hypomotility (lower *δ*) or decreased gastric tone (lower *p*_*o*_). The size and shade of the circles scale with the corresponding quantity in each figure. (*a*) Pre-op. net emptying rate (ml min^−1^), (*b*) post-op. net emptying rate (ml min^−1^), (*c*) post-op. reflux (ml min^−1^).

So far, we have separately considered cases of extreme hypomotility or decreased gastric tone. We now use the model to examine how the emptying rate and the efficacy of pyloric enlargement vary for mild occurrence or for a concomitant occurrence of these mechanisms in a gastroparesis patient. Figure 11 shows the pre-operative emptying rate, post-operative emptying rate and the amount of post-operative bile reflux, on the motility-tone chart. The *x*-axes of these plots go from extreme antral hypomotility (*δ* = 0.2) to healthy (*δ* = 0.4), and the *y*-axes go from extremely low gastric tone (*p*_*o*_ = 0) to healthy (*p*_*o*_ = 0.4) for a meal with a viscosity of 1 mPa s.

For the healthy case (control) case, the combined action of the gastric tone and the ACW results in an emptying rate of 4.3 ml min^−1^, as shown in figure 11*a*. On the opposite spectrum is the concomitant extreme occurrence of both mechanisms (i.e. *δ* = 0.2 and *p*_*o*_ = 0), which, as expected, leads to the lowest emptying rate, which is only about 44% of the control. Severe reduction in gastric tone alone (*δ* = 0.4 and *p*_*o*_ = 0.0) results in a reduction of gastric emptying to 65% its nominal value. On the other hand, reduction in antral motility with preserved gastric tone results in a reduction of emptying rate to 84% from the nominal value. The pre-operative emptying rate for the mild concomitant occurrence of both mechanisms (*δ* = 0.3 and *p*_*o*_ = 0.02) lies between that of the extreme occurrence of each mechanism. All these cases exhibit a reduction in emptying rate that is typical of gastroparesis patients. In one study, the average half-emptying time (i.e. the time it takes to empty half of the contents) of diabetic gastroparesis patients was found to be 132 ± 17 min while that of controls was 62 ± 11 min, which corresponds to the patient’s emptying rate becoming almost half of the healthy cases [48].

Post-operatively, as shown in figure 11*b*, the procedure shows the maximum improvement in emptying rate for the hypomotility case with preserved gastric tone (by 131%). Such post-procedural improvements have been observed by surgeons—Hibbard *et al.* [20] noted that the mean half-emptying time of patients decreased from 320 to 112 min, which corresponds to the emptying rate being doubled after the surgery; and Mancini *et al.* [12] reported a drop in the same from 242.7 to 106.5 min, which implies an even larger improvement in the emptying rate. For the phenotypes with severely reduced gastric tone, figure 11*b* shows that the net emptying rate reduces almost to zero, irrespective of the antral motility. The case with a modest reduction in gastric tone (*p*_*o*_ = 0.02) recovers a nearly normal emptying rate post-procedurally. This observation highlights that while both phenotypes of gastroparesis modelled here exhibit a similar pre-operative reduction in emptying rates, their post-operative response is significantly different. Figure 11*c* reveals the reason behind the low post-operative emptying rate of low gastric tone cases after the procedure—they have large amounts of bile reflux as compared with other cases. Interestingly, since the reflux occurs during the relaxation phase of the contraction after the TAC, pre-operative bile reflux *decreases* with a reduction in antral motility for a fixed gastric tone, due to smaller changes in volume during a weaker TAC.

This suggests that not all gastroparesis patients benefit equally from pyloric enlargement procedures. If the reduction in emptying rate is due to reduced hypomotility and the gastric tone is retained, even partially, then the procedure can be very effective. Indeed, the procedure may even increase the emptying rate significantly beyond nominal values for some patients, leading to dumping syndrome [4,55].

These observations align with the long-established understanding that the proximal stomach is primarily responsible for the emptying of meals [2]. Researchers have also found that increased intragastric pressure increases the emptying rate [50], and, conversely, the use of fundus relaxing drugs slows it down [56].

### 3.5. Effect of increasing post-operative orifice diameters

**Figure 12.**
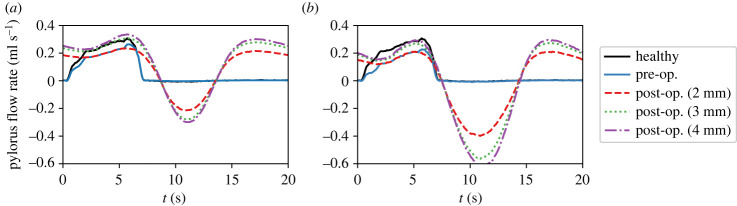
The flow rate through the pylorus is shown for different post-operative orifice diameters in each condition. In the fed state, the pre-operative diameter was 2 mm, and after the procedure, three different scenarios are considered—a diameter of 2, 3 and 4 mm. Larger diameters result in larger improvements in emptying rates but also lead to increased bile reflux. (*a*) Antral hypomotility and (*b*) decreased gastric tone.

In all the results discussed above, the post-operative case corresponded to an enlargement (*E*) of 2 mm. We also saw that while the procedure can improve the emptying rate it can also lead to reflux from the duodenum into the stomach. It is expected that these features would be sensitive to the degree of enlargement achieved during the surgery. We have therefore simulated two additional post-operative cases with higher values of *E*, and figure 12 shows the flux through the pylorus before and after the procedure for all three post-operative orifice diameters.

**Figure 13.**
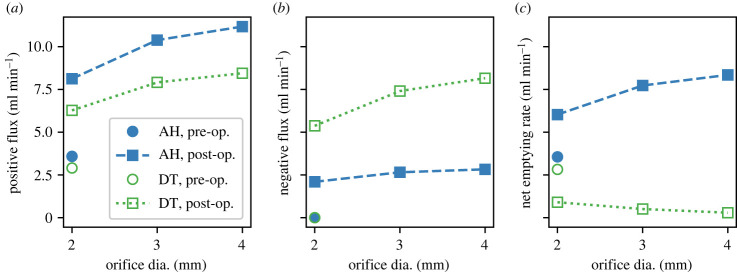
The figure shows the rate of positive flux, negative flux (i.e. reflux), and the net emptying rate for different post-operative orifice diameters. The net emptying rate is calculated by integrating the pyloric flux over the entire duration, while the positive and the negative flux are calculated by integrating only over the duration for which the flow is anterograde (from the stomach into the duodenum) and retrograde (from the duodenum into the stomach), respectively.

Figure 13 quantifies this sensitivity towards post-operative orifice diameter. For the hypomotility case, the net emptying rate improves more when going from 2 to 3 mm as compared with that from 3 to 4 mm. For the reduced gastric tone phenotype, on the other hand, the net emptying rate reduces with an increase in orifice enlargement. The amount of reflux also rises with increasing pyloric enlargement, but the reflux for decreased tone cases is much larger, which is the reason behind their low post-operative emptying rates. Firstly, this emphasizes the huge gap in the post-operative behaviour of the two mechanisms. Secondly, it shows that even for cases that do show improvement, in order to achieve a desired correction in the emptying rate, a specific pyloric orifice diameter should be targeted that is determined based on the extent of reduction in the emptying rate of that patient.

## 4. Conclusion

We used a computational model of the stomach to model gastroparesis—a disorder that reduces the rate of gastric emptying in the absence of any mechanical obstruction. We account for two phenotypes of gastroparesis—antral hypomotility and decreased gastric tone. The former was modelled as reduced contractions of the stomach walls, and the latter as a reduction in pressure applied by the upper part of the stomach on the contents. After comparing our model with existing *in vivo* measurements, we simulated the two phenotypes occurring separately as well as concomitantly. We found that both manifestations of gastroparesis independently lead to a reduction in the emptying rate of the stomach. The hypomotility case shows weaker retrograde jets while the decreased tone case has a weaker pyloric jet.

The effect of pyloroplasty procedures on gastroparesis cases is studied next. This procedure enlarges the pyloric orifice—the exit of the stomach that connects it to the small intestines—and disrupts its sphincter function with the goal of facilitating emptying and ameliorating gastroparesis symptoms. Our model indicates that the hypomotility phenotype benefits more from these procedures than cases with decreased gastric tone. When both conditions occur simultaneously, the procedure is helpful only if the stomach retains some of its gastric tone. If the stomach is unable to generate the necessary tonic pressure on its contents, then enlarging the orifice diameter does not improve the emptying rate, but in fact, reduces it further because of the increased level of reflux from the duodenum into the stomach.

The findings demonstrate a scenario where two distinct phenotypes of gastroparesis presenting with similar reductions in gastric emptying, respond very differently to pyloroplasty, presenting a possible explanation for the uneven response of patients to these pyloric intervention procedures [13]. There is increasing interest in pre-operative tests that can indicate whether a patient will respond to these surgeries [57], and it is expected that in the future, physiological measurements will be used to optimize the choice of therapy [50]. We demonstrate the ability of computational models to contribute to our understanding of gastric disorders and surgeries.

It is worth pointing out that the model fails to account for the duodenal feedback that would regulate gastric emptying, especially after a large increase in emptying rate after the procedure. We assume that the gastric tone does not change after the procedure for the same meal. Furthermore, we have only considered liquid meals in this work. Although we do not expect the emptying rate conclusions to change significantly in the presence of solid foods, the hypomotility cases would suffer additionally from slower physical breakdown of solid particles. We are currently working on extending this model to incorporate solid meals. However, withstanding these limitations, the model still generates novel insights into gastroparesis and helps understand the changes in the flow field due to pyloric intervention procedures.

## Data Availability

The data and model can be accessed from the GitHub repository: https://github.com/sharunkuhar/insilico_stomachsim_pyloroplasty [58]. The stomach geometry is provided in electronic supplementary material [59].

## References

[1] Hellström PM, Grybäck P, Jacobsson H. 2006 The physiology of gastric emptying. Best Pract. Res. Clin. Anaesthesiol. **20**, 397-407. (10.1016/j.bpa.2006.02.002) 17080692

[2] Kelly KA. 1980 Gastric emptying of liquids and solids: roles of proximal and distal stomach. Am. J. Physiol.-Gastrointest. Liver Physiol. **239**, G71-G76. (10.1152/ajpgi.1980.239.2.G71) 6996495

[3] Urbain JL, Siegel JA, Charkes N, Maurer AH, Malmud LS, Fisher RS. 1989 The two-component stomach: effects of meal particle size on fundal and antral emptying. Eur. J. Nuclear Med. **15**, 254-259. (10.1007/BF00257543) 2759125

[4] Ukleja A. 2005 Dumping syndrome: pathophysiology and treatment. Nutr. Clin. Pract. **20**, 517-525. (10.1177/0115426505020005517) 16207692

[5] Camilleri M, Kuo B, Nguyen L, Vaughn VM, Petrey J, Greer K, Yadlapati R, Abell TL. 2022 ACG clinical guideline: gastroparesis. Am. J. Gastroenterol. **117**, 1197-1220. (10.14309/ajg.0000000000001874) 35926490 PMC9373497

[6] Hardoff R, Sula M, Tamir A, Soil A, Front A, Badarna S, Honigman S, Giladi N. 2001 Gastric emptying time and gastric motility in patients with Parkinson’s disease. Mov. Disord. **16**, 1041-1047. (10.1002/mds.1203) 11748735

[7] Martinek J et al. 2022 Endoscopic pyloromyotomy for the treatment of severe and refractory gastroparesis: a pilot, randomised, sham-controlled trial. Gut **71**, 2170-2178. (10.1136/gutjnl-2022-326904) 35470243 PMC9554080

[8] Grover M, Farrugia G, Stanghellini V. 2019 Gastroparesis: a turning point in understanding and treatment. Gut **68**, 2238-2250. (10.1136/gutjnl-2019-318712) 31563877 PMC6874806

[9] Camilleri M, Parkman HP, Shafi MA, Abell TL, Gerson L. 2013 Clinical guideline: management of gastroparesis. Am. J. Gastroenterol. **108**, 18-37. (10.1038/ajg.2012.373) 23147521 PMC3722580

[10] Wadhwa V, Mehta D, Jobanputra Y, Lopez R, Thota PN, Sanaka MR. 2017 Healthcare utilization and costs associated with gastroparesis. World J. Gastroenterol. **23**, 4428. (10.3748/wjg.v23.i24.4428) 28706426 PMC5487507

[11] Yu D, Ramsey FV, Norton WF, Norton N, Schneck S, Gaetano T, Parkman HP. 2017 The burdens, concerns, and quality of life of patients with gastroparesis. Dig. Dis. Sci. **62**, 879-893. (10.1007/s10620-017-4456-7) 28110376

[12] Mancini SA, Angelo JL, Peckler Z, Philp FH, Farah KF. 2015 Pyloroplasty for refractory gastroparesis. Am. Surg. **81**, 738-746. (10.1177/000313481508100726) 26140897

[13] Ramos GP, Camilleri M. 2023 Ten controversies in gastroparesis and a look to the future. Neurogastroenterol. Motility **35**, e14494. (10.1111/nmo.14494) PMC10133001 36371704

[14] Vosoughi K et al. 2020 Role of endoscopic functional luminal imaging probe in predicting the outcome of gastric peroral endoscopic pyloromyotomy (with video). Gastrointest. Endosc. **91**, 1289-1299. (10.1016/j.gie.2020.01.044) 32035074

[15] Camilleri M. 2016 Novel diet, drugs, and gastric interventions for gastroparesis. Clin. Gastroenterol. Hepatol. **14**, 1072-1080. (10.1016/j.cgh.2015.12.033) 26762845 PMC4931993

[16] Mohan BP. 2020 Clinical efficacy of gastric per-oral endoscopic myotomy (G-POEM) in the treatment of refractory gastroparesis and predictors of outcomes: a systematic review and meta-analysis using surgical pyloroplasty as a comparator group. Surg. Endosc. **34**, 3352-3367. (10.1007/s00464-019-07135-9) 31583465

[17] Park PO, Bergström M, Ikeda K, Fritscher-Ravens A, Mosse S, Kochman M, Swain P. 2007 Endoscopic pyloroplasty with full-thickness transgastric and transduodenal myotomy with sutured closure. Gastrointest. Endosc. **66**, 116-120. (10.1016/j.gie.2006.10.018) 17451701

[18] Landreneau JP, Strong AT, El-Hayek K, Tu C, Villamere J, Ponsky JL, Kroh MD, Rodriguez JH. 2019 Laparoscopic pyloroplasty versus endoscopic per-oral pyloromyotomy for the treatment of gastroparesis. Surg. Endosc. **33**, 773-781. (10.1007/s00464-018-6342-6) 30019220

[19] Aghaie Meybodi M, Qumseya B, Shakoor D, Lobner K, Vosoughi K, Ichkhanian Y, Khashab M. 2019 Efficacy and feasibility of G-POEM in management of patients with refractory gastroparesis: a systematic review and meta-analysis. Endoscopy Int. Open **7**, E322-E329. (10.1055/a-0812-1458) PMC6400657 30842971

[20] Hibbard ML, Dunst CM, Swanström LL. 2011 Laparoscopic and endoscopic pyloroplasty for gastroparesis results in sustained symptom improvement. J. Gastrointest. Surg. **15**, 1513-1519. (10.1007/s11605-011-1607-6) 21720926

[21] Brough WA, Taylor TV, Torrance HB. 1984 The surgical factors influencing duodenogastric reflux. Brit. J. Surg. **71**, 770-773. (10.1002/bjs.1800711011) 6487975

[22] Eriksson B, Szegö T, Emås S. 1990 Duodenogastric bile reflux before and after selective proximal vagotomy with and without pyloroplasty. Scand. J. Gastroenterol. **25**, 161-164. (10.3109/00365529009107938) 2406891

[23] Roses RE, Fraker DL. 2015 Bile reflux and gastroparesis. In *Gastrointestinal surgery: management of complex perioperative complications* (eds TM Pawlik, SK Maithel, NB Merchant), pp. 119–125. New York, NY: Springer.

[24] Pal A, Indireshkumar K, Schwizer W, Abrahamsson B, Fried M, Brasseur JG. 2004 Gastric flow and mixing studied using computer simulation. Proc. R. Soc. Lond. B **271**, 2587-2594. (10.1098/rspb.2004.2886) PMC1691895 15615685

[25] Kozu H, Kobayashi I, Nakajima M, Uemura K, Sato S, Ichikawa S. 2010 Analysis of flow phenomena in gastric contents induced by human gastric peristalsis using CFD. Food Biophys. **5**, 330-336. (10.1007/s11483-010-9183-y) 24931649

[26] Alokaily S, Feigl K, Tanner FX. 2019 Characterization of peristaltic flow during the mixing process in a model human stomach. Phys. Fluids **31**, 103105. (10.1063/1.5122665)

[27] Singh SK. 2007 *Fluid flow and disintegration of food in human stomach*. PhD thesis, University of California, Davis, CA.

[28] Ferrua M, Singh R. 2010 Modeling the fluid dynamics in a human stomach to gain insight of food digestion. J. Food Sci. **75**, R151-R162. (10.1111/j.1750-3841.2010.01748.x) 21535567 PMC2992692

[29] Imai Y, Kobayashi I, Ishida S, Ishikawa T, Buist M, Yamaguchi T. 2013 Antral recirculation in the stomach during gastric mixing. Am. J. Physiol.-Gastrointest. Liver Physiol. **304**, G536-G542. (10.1152/ajpgi.00350.2012) 23275619

[30] Ishida S, Miyagawa T, O’Grady G, Cheng LK, Imai Y. 2019 Quantification of gastric emptying caused by impaired coordination of pyloric closure with antral contraction: a simulation study. J. R. Soc. Interface **16**, 20190266. (10.1098/rsif.2019.0266) 31387481 PMC6731493

[31] Li C, Jin Y. 2021 A CFD model for investigating the dynamics of liquid gastric contents in human-stomach induced by gastric motility. J. Food Eng. **296**, 110461. (10.1016/j.jfoodeng.2020.110461)

[32] Li C, Xiao J, Chen XD, Jin Y. 2021 Mixing and emptying of gastric contents in human-stomach: a numerical study. J. Biomech. **118**, 110293. (10.1016/j.jbiomech.2021.110293) 33588327

[33] Acharya S, Halder S, Kou W, Kahrilas PJ, Pandolfino JE, Patankar NA. 2022 A fully resolved multiphysics model of gastric peristalsis and bolus emptying in the upper gastrointestinal tract. Comput. Biol. Med. **143**, 104948. (10.1016/j.compbiomed.2021.104948) 35091365 PMC9014465

[34] Kuhar S, Lee JH, Seo JH, Pasricha PJ, Mittal R. 2022 Effect of stomach motility on food hydrolysis and gastric emptying: insight from computational models. Phys. Fluids **34**, 111909. (10.1063/5.0120933) PMC9667910 36407285

[35] Seo JH, Mittal R. 2022 Computational modeling of drug dissolution in the human stomach. Front. Physiol. **12**, 755997. (10.3389/fphys.2021.755997) 35082685 PMC8785969

[36] Lee JH, Kuhar S, Seo JH, Pasricha PJ, Mittal R. 2022 Computational modeling of drug dissolution in the human stomach: effects of posture and gastroparesis on drug bioavailability. Phys. Fluids **34**, 081904. (10.1063/5.0096877) PMC9372820 35971381

[37] Gosselin MC et al. 2014 Development of a new generation of high-resolution anatomical models for medical device evaluation: the virtual population 3.0. Phys. Med. Biol. **59**, 5287-5303. (10.1088/0031-9155/59/18/5287) 25144615

[38] Rao S, Lu C, Schulze-Delrieu K. 1996 Duodenum as a immediate brake to gastric outflow: a videofluoroscopic and manometric assessment. Gastroenterology **110**, 740-747. (10.1053/gast.1996.v110.pm8608883) 8608883

[39] Bornhorst GM, Paul Singh R. 2014 Gastric digestion *in vivo* and *in vitro*: how the structural aspects of food influence the digestion process. Annu. Rev. Food Sci. Technol. **5**, 111-132. (10.1146/annurev-food-030713-092346) 24387607

[40] Schwizer W, Fraser R, Borovicka J, Asal K, Crelier G, Kunz P, Boesiger P, Fried M. 1996 Measurement of proximal and distal gastric motility with magnetic resonance imaging. Am. J. Physiol.-Gastrointest. Liver Physiol. **271**, G217-G222. (10.1152/ajpgi.1996.271.1.G217) 8760126

[41] Kunz P, Feinle C, Schwizer W, Fried M, Boesiger P. 1999 Assessment of gastric motor function during the emptying of solid and liquid meals in humans by MRI. J. Magn. Reson. Imaging **9**, 75-80. (10.1002/(SICI)1522-2586(199901)9:1<75::AID-JMRI10>3.0.CO;2-I) 10030653

[42] Marciani L, Young P, Wright J, Moore R, Coleman N, Gowland PA, Spiller RC. 2001 Antral motility measurements by magnetic resonance imaging. Neurogastroenterol. Motil. **13**, 511-518. (10.1046/j.1365-2982.2001.00285.x) 11696113

[43] Marciani L, Gowland PA, Fillery-Travis A, Manoj P, Wright J, Smith A, Young P, Moore R, Spiller RC. 2001 Assessment of antral grinding of a model solid meal with echo-planar imaging. Am. J. Physiol.-Gastrointest. Liver Physiol. **280**, G844-G849. (10.1152/ajpgi.2001.280.5.G844) 11292591

[44] Kwiatek MA et al. 2009 Effect of meal volume and calorie load on postprandial gastric function and emptying: studies under physiological conditions by combined fiber-optic pressure measurement and MRI. Am. J. Physiol.-Gastrointest. Liver Physiol. **297**, G894-G901. (10.1152/ajpgi.00117.2009) 19779010

[45] Palmada N, Hosseini S, Avci R, Cater JE, Suresh V, Cheng LK. 2023 A systematic review of computational fluid dynamics models in the stomach and small intestine. Appl. Sci. **13**, 6092. (10.3390/app13106092)

[46] Brener W, Hendrix TR, McHugh PR. 1983 Regulation of the gastric emptying of glucose. Gastroenterology **85**, 76-82. (10.1016/S0016-5085(83)80232-7) 6852464

[47] Marciani L, Gowland PA, Spiller RC, Manoj P, Moore RJ, Young P, Fillery-Travis AJ. 2001 Effect of meal viscosity and nutrients on satiety, intragastric dilution, and emptying assessed by MRI. Am. J. Physiol.-Gastrointest. Liver Physiol. **280**, G1227-G1233. (10.1152/ajpgi.2001.280.6.G1227) 11352816

[48] Urbain JLC et al. 1993 Characterization of gastric antral motility disturbances in diabetes using a scintigraphic technique. J. Nucl. Med. **34**, 576-581. 8455073

[49] Camilleri M, Sanders KM. 2022 Gastroparesis. Gastroenterology **162**, 68-87.e1. (10.1053/j.gastro.2021.10.028) 34717924 PMC8678360

[50] Camilleri M. 2021 Relationship of motor mechanisms to gastroparesis symptoms: toward individualized treatment. Am. J. Physiol.-Gastrointest. Liver Physiol. **320**, G558-G563. (10.1152/ajpgi.00006.2021) 33566731 PMC8238170

[51] Camilleri M, Bharucha AE, Farrugia G. 2011 Epidemiology, mechanisms, and management of diabetic gastroparesis. Clin. Gastroenterol. Hepatol. **9**, 5-12. (10.1016/j.cgh.2010.09.022) 20951838 PMC3035159

[52] Indireshkumar K et al. 2000 Relative contributions of ‘pressure pump’ and ‘peristaltic pump’ to gastric emptying. Am. J. Physiol.-Gastrointest. Liver Physiol. **278**, G604-G616. (10.1152/ajpgi.2000.278.4.G604) 10762615

[53] Malbert CH, Ruckebusch Y. 1991 Relationships between pressure and flow across the gastroduodenal junction in dogs. Am. J. Physiol.-Gastrointest. Liver Physiol. **260**, G653-G657. (10.1152/ajpgi.1991.260.4.G653) 2018139

[54] Malbert CH, Serthelon JP, Dent J. 1992 Changes in antroduodenal resistance induced by cisapride in conscious dogs. Am. J. Physiol.-Gastrointest. Liver Physiol. **263**, G202-G208. (10.1152/ajpgi.1992.263.2.G202) 1514631

[55] Tack J, Arts J, Caenepeel P, De Wulf D, Bisschops R. 2009 Pathophysiology, diagnosis and management of postoperative dumping syndrome. Nat. Rev. Gastroenterol. Hepatol. **6**, 583-590. (10.1038/nrgastro.2009.148) 19724252

[56] Tack J, Piessevaux H, Coulie B, Caenepeel P, Janssens J. 1998 Role of impaired gastric accommodation to a meal in functional dyspepsia. Gastroenterology **115**, 1346-1352. (10.1016/S0016-5085(98)70012-5) 9834261

[57] Malik Z, Kataria R, Modayil R, Ehrlich AC, Schey R, Parkman HP, Stavropoulos SN. 2018 Gastric per oral endoscopic myotomy (G-POEM) for the treatment of refractory gastroparesis: early experience. Dig. Dis. Sci. **63**, 2405-2412. (10.1007/s10620-018-4976-9) 29468376

[58] Kuhar S, Seo J-H, Pasricha PJ, Mittal R. 2024 Data from: *In silico* modelling of the effect of pyloric intervention procedures on gastric flow and emptying in a stomach with gastroparesis. GitHub repository. (https://github.com/sharunkuhar/insilico_stomachsim_pyloroplasty) 10.1098/rsif.2023.0567 PMC10824103 38263890

[59] Kuhar S, Seo J-H, Pasricha PJ, Mittal R. 2024 *In silico* modelling of the effect of pyloric intervention procedures on gastric flow and emptying in a stomach with gastroparesis. Figshare. (10.6084/m9.figshare.c.7020985) PMC10824103 38263890

